# A preliminary study of the effect of a high-salt diet on transcriptome dynamics in rat hypothalamic forebrain and brainstem cardiovascular control centers

**DOI:** 10.7717/peerj.8528

**Published:** 2020-03-03

**Authors:** Chitra Devi Ramachandran, Khadijeh Gholami, Sau Kuen Lam, See Ziau Hoe

**Affiliations:** 1 Department of Physiology, Faculty of Medicine, University of Malaya, Kuala Lumpur, Wilayah Perseketuan, Malaysia; 2 Human Biology Division, School of Medicine, International Medical University, Kuala Lumpur, Wilayah Perseketuan, Malaysia; 3 Department of Pre-Clinical Sciences, Faculty of Medicine and Health Sciences, Universiti Tunku Abdul Rahman, Sungai Long, Selangor, Malaysia

**Keywords:** High-salt diet, Nucleus tractus solitarii (NTS), Hypertension, Subfornical organ (SFO), Rostral ventrolateral medulla (RVLM), Paraventricular nucleus (PVN), Supraoptic nuclei (SON)

## Abstract

**Background:**

High dietary salt intake is strongly correlated with cardiovascular (CV) diseases and it is regarded as a major risk factor associated with the pathogenesis of hypertension. The CV control centres in the brainstem (the nucleus tractus solitarii (NTS) and the rostral ventrolateral medulla (RVLM)) and hypothalamic forebrain (the subfornical organ, SFO; the supraoptic nucleus, SON and the paraventricular nucleus, PVN) have critical roles in regulating CV autonomic motor outflows, and thus maintaining blood pressure (BP). Growing evidence has implicated autonomic regulatory networks in salt-sensitive HPN (SSH), but the genetic basis remains to be delineated. We hypothesized that the development and/ or maintenance of SSH is reliant on the change in the expression of genes in brain regions controlling the CV system.

**Methodology:**

We used RNA-Sequencing (RNA-Seq) to describe the differential expression of genes in SFO, SON, PVN, NTS and RVLM of rats being chronically fed with high-salt (HS) diet. Subsequently, a selection of putatively regulated genes was validated with quantitative reverse transcription polymerase chain reaction (qRT-PCR) in both Spontaneously Hypertensive rats (SHRs) and Wistar Kyoto (WKY) rats.

**Results:**

The findings enabled us to identify number of differentially expressed genes in SFO, SON, PVN, NTS and RVLM; that are either up-regulated in both strains of rats (SON- *Caprin2*, *Sctr*), down-regulated in both strains of rats (PVN- *Orc*, *Gkap1*), up-regulated only in SHRs (SFO- *Apopt1*, *Lin52*, *AVP*, *OXT*; SON- *AVP*, *OXT*; PVN- *Caprin2*, *Sclt;* RVLM- *A4galt*, *Slc29a4*, *Cmc1*) or down-regulated only in SHRs (SON- *Ndufaf2*, *Kcnv1*; PVN- *Pi4k2a*; NTS- *Snrpd2l*, *Ankrd29*, *St6galnac6*, *Rnf157*, *Iglon5*, *Csrnp3*, *Rprd1a*; RVLM- *Ttr*, *Faim*).

**Conclusions:**

These findings demonstrated the adverse effects of HS diet on BP, which may be mediated via modulating the signaling systems in CV centers in the hypothalamic forebrain and brainstem.

## Introduction

Hypertension (HPN) results from a complex interaction between genetic predisposition and environmental factors such as dietary salt intake. Dietary salt (i.e., sodium chloride, NaCl) intake is the most remarkable modifiable environmental factor that attracts many studies in HPN. Indeed, it has been acknowledged as an important contributing factor of the etiology and progression of HPN (Johnson et al., 2015). A marked elevation in BP in response to excessive dietary salt intake, known as salt-sensitive HPN (SSH), has been observed in humans as well as experimental animals (Guyenet, 2006a; Iwamoto, Kita & Katsuragi, 2005). Despite the abundant experimental, interventional and epidemiological observations demonstrating an association between dietary salt and HPN, mechanisms linking high-salt (HS) intake to the increase in BP are not well understood.

High-salt intake has been reported to influence the excitability of sympathetic regulatory networks which the brain regions controlling sympathetic tone play an important role in SSH (Carmichael et al., 2015; Guo et al., 2015; Orlov & Mongin, 2007). Studies have shown that blockade of sympathetic outflow; and transection of sympathetic nerves consistently lowers arterial BP, while lesions of anteroventral third ventricular regions prevent or attenuates the development and/or the severity of SSH (Johnson et al., 2015; King, Osborn & Fink, 2007; Stocker, Madden & Sved, 2010). Furthermore, there are now unequivocal evidences that central brain regions regulating CV autonomic motor outflows are sensitive to salt. These brain regions regulate sympathetic activity and their resultant over activity triggered by HS diet is associated with the development, maintenance and progression of hypertensive state in human patients and animal models (Esler & Kaye, 2000; Guyenet, 2006a; Lohmeier, 2002; Mann, 2003; Petersson et al., 2002; Sakata et al., 2002).

The application of newly developed neuroanatomical and functional techniques, allowed to identify neural network comprising key brain nuclei and their interconnections that controls BP (Lohmeier et al., 2010; Thrasher, 2002; Thrasher, 2006). Both the hypothalamic forebrain and brainstem are regarded as essential components in the regulatory neuronal network of central BP control. The regions in the hypothalamic forebrain act as an interface between the endocrine and nervous system and a wide variety of functional changes in it characterize multiple forms of HPN including SSH (Carmichael & Wainford, 2015). The brainstem, on the other hand, plays an important role in the control of cardiac and vascular baroreceptor reflexes to regulate arterial BP (Sved, Ito & Sved, 2003).

In the present study, one of the circumventricular organs (CVOs)—the subfornical organ (SFO) which is known to be important in regulating fluid homeostasis and drinking behaviors in SSH; two hypothalamic forebrain regions that are known for regulating osmotic stability in SSH—the supraoptic nucleus (SON) and the paraventricular nucleus (PVN); and two important medullary structures—the nucleus tractus solitarii (NTS) and the rostral ventrolateral medulla (RVLM), which both play important roles in the control of cardiac and vascular baroreceptor reflexes in SSH have been focused.

The SFO which is situated on the midline wall of the third ventricle in the dorsal region of lamina teminalis is endowed with numbers of functional receptors for signals vital in the regulation of fluid homeostasis and drinking behaviors (Ahmed et al., 2014). In addition, the neurons of the SFO exert their effects by direct and indirect efferent projections to other important hypothalamic nuclei that are involved in controlling autonomic functions or neuroendocrine actions including PVN and the SON (Cottrell & Ferguson, 2004; Rowland et al., 1996; Smith & Ferguson, 2010); as well as to key CV regulatory brainstem sites, NTS and the RVLM (Moellenhoff et al., 1998; Sakai et al., 2007). Meanwhile, both PVN and SON at the hypothalamic forebrain are known as important integrative structures that regulate coordinated responses to perturbations in CV homeostasis through endocrine projections especially in the regulation of osmotic stability (Hindmarch et al., 2006). The PVN, in addition, has been reported to reciprocally projects to autonomic nuclei in brainstem (NTS and RVLM) and spinal cord which are responsible in activating sympathetic nervous system (SNS) including CV regulation (Pyner, 2009). Here, they have the potential to change sympathetic nerves activity by virtue of direct descending projections that terminate on or near to sympathetic pre-ganglionic neurones found in intermediolateral cell column (IML) of the spinal cord (Motawei et al., 1999; Pyner, 2009; Ranson et al., 1998).

The NTS, on the other hand, plays a pivotal role in the regulation of both the set-point and the gain of baroreflex for homeostatic control of BP (Waki et al., 2010; Waki, Takagishi & Gouraud, 2014). It is the principal site of termination of baroreceptor afferent fibers and as such mediate inhibitory action of baroreceptor on sympathetic discharge (Frigero, Bonagamba & Machado, 2000; Machado, 2001; Waki, Takagishi & Gouraud, 2014). This area contains many neurotransmitters or neuromodulators that are important in CV control, and the intermediate portion of NTS is richly innervated by fibers arising from different brain nuclei that are also known to have an important role in CV control (Colombari et al., 2001; Duale et al., 2007; Kasparov & Teschemacher, 2008; Zoccal et al., 2014). The NTS neurons send excitatory amino acid projections to the caudal ventrolateral medulla (CVLM) which, in turn, inhibits RVLM neurons via GABAergic inhibitory pathway (Guyenet, 2006b; Sapru, 1996; Yao et al., 2008). Meanwhile, RVLM lies ventral to the rostral part of the nucleus ambiguous, caudal to the facial nucleus and ventral to Bötzinger complex (Dampney, 1994; Geraldes et al., 2014b). The RVLM is the region where the sympathetic pre-motor neurons controlling vasomotor sympathetic nerve activity are located. The RVLM neurons project to the sympathetic preganglionic neurons in the IML cell column of the spinal cord and receive a direct glutamatergic projection from NTS (Card et al., 2006; Dampney, 1994; Geraldes et al., 2014b; Leman, Viltart & Sequeira, 2000). These neurons also receive excitatory and inhibitory inputs from other brain areas such as hypothalamus and other part of medulla oblongata (Koga et al., 2008; Sved, Ito & Yajima, 2002).

Hence, the brain mechanisms in both initiation and maintenance of BP are explored as increasing evidence as a key contributor to the development and maintenance of SSH (Carmichael & Wainford, 2015; DiBona, 2013).

In the present study, we hypothesized that elevated dietary salt intake might alter gene expression in the SFO, SON, PVN, NTS and RVLM which may affect the functional activity of these nuclei, resulting in altered excitability and increases the gain of central sympathetic neurons. Hence, we have used Next Generation Sequencing (RNA-Seq) to describe the transcriptional profile of the hypothalamic forebrain regions of SFO, SON and PVN; and the medullary NTS and RVLM of SHRs under condition of regular- and high-salt consumptions. Comparison of these datasets has allowed us to identify genes that are putatively differentially expressed as a result of a chronic HS diet. We have further validated these data using quantitative reverse transcription PCR (qRT-PCR) in the SHR, and in the WKY rats.

## Materials and Methods

### Animals and animal care

The male WKY rats and SHRs used in this study were bred at the University of Malaya Animal Experimental Unit from stock obtained from BioLASCO (Taiwan). After being weaned at five weeks of age, rats were housed in groups of five to six under controlled laboratory conditions (temperature 23 ± 5 °C, 12:12-hour light/ dark cycle and humidity 50% to 60%) with food and water *ad libitum* for at least a week prior to the onset of experimentation. All the experimental protocols involving animals and housing thereof were reviewed and approved by the Institutional Animal Care and Use Committee (IACUC) of the University of Malaya (Reference: 2014-01-07/Physio/R/HSZ) which maintains a full Association for Assessment and Accreditation of Laboratory Animal Care (AAALAC) accreditation.

### Grouping and experimental design

Six-week-old WKY rats and SHRs were randomly assigned to receive food with either a regular-salt (RS) content (0.2% w/v NaCl) or high-salt (HS) content (4% w/v NaCl; Harlan Teklad, Germany) with free access of water. Four groups were thus studied:

Group 1: WKY rats receiving RS diet (WRS)

Group 2: WKY rats receiving HS diet (WHS)

Group 3: SHRs receiving RS diet (SRS)

Group 4: SHRs receiving HS diet (SHS)

The treatment period continued for six weeks.

### Tissue collection and preparation

At the end of diet treatment, rats were euthanized (between the hours of 0800 to 1100) and tissue was isolated and processed as described below. Rats were humanely killed by stunning, followed immediately by decapitation with an animal guillotine (Harvard Apparatus, Holliston, MMA, USA). Brains were quickly removed from the cranium, washed in PBS, then cut into two parts to separate the forebrain and the brainstem which were then snap-frozen in dry ice (within 3 min after stunning) before being stored at −80 °C. The SFO, SON, PVN, NTS and RVLM were accurately localised based on rat brain atlas (George Paxinos & Watson, 2014). Frozen forebrain and brainstem were mounted on the cryostat stage set at −20 °C, equilibrated for 10 min and sectioned at 60 µm using a Thermo Scientific Shandon Cryotome FE and FSEE Cryostat. The sections were mounted on glass slides and stained with Toluidine blue (Sigma Aldrich; 1% w/v in 70% v/v ethanol) then visualised on a light microscope to identify the SFO, SON, PVN (at forebrain) and NTS and RVLM (at brainstem). Once localised, punches of SFO, SON, PVN, NTS and RVLM were then taken with one mm and 0.5 mm micro punches (Fine Scientific Tools) from the unstained tissue. The SFO was collected upon identification of anterior commissure followed by choroid plexus meets the third ventricle to form interventricular foremen. Six to eight consecutive punches were made to collect SFO region. Meanwhile, the supraoptic chiasm which was observance by ‘naked’ eye was used as reference point to collect SON and 12 to 14 serial punches were made to collect this region as defined by the rat brain atlas. Meanwhile, about eight consecutive punches were made upon opening of third ventricle to collect PVN. On the other hand, hypoglossal nucleus and facial nucleus were used as references for dissecting NTS (caudal, intermediate and rostral) and RVLM, respectively. As defined by the rat brain atlas, 20 to 23 consecutive sections were made for NTS, with the first six slices being central punches and the rest bilateral punches. For the RVLM, eight consecutive sections were punched after the disappearance of the facial nucleus. Each of the SFO, SON, PVN, NTS and RVLM punches then dispensed into 1.5ml tubes, suspended QIAzol reagent then stored at −80 °C prior to RNA extraction.

### RNA extraction and quality assessment

Total RNA was extracted using Qiagen RNeasy kit protocols (Qiagen, Hilden, Germany). The frozen punched samples were allowed to thaw to ambient temperature and QIAzol phase later was separated with 350 µl chloroform (15 min, 12,000 g, 4 °C). The upper aqueous phase was removed then mixed with 70% (v/v) ethanol to precipitate the total RNA, which was resuspended and applied to RNeasy columns in accordance with the manufacturer’s instruction. For RNAseq experiments, punched samples at QIAzol phase were pooled (5 per group), whereas individual samples were used in qRT-PCR (*n* = 6). The RNA concentration was measured using a Nanodrop spectrophotometer (ND-2000, Thermo Scientific, Wilmington, DE) and Qubit Fluorometer 2.0 (Life Technologies). The RNA samples were also analysed using 2100 Bioanalyzer (Agilent Technologies, Santa Clara, CA 95051) to obtain RNA integrity numbers (RIN numbers) as a measure of their quality (Schroeder et al., 2006). All RNA samples met the RIN quality criterion of >8.5.

### RNA-Sequencing analysis

Amplified cDNA libraries were prepared from isolated SFO, SON, PVN, NTS and RVLM RNA samples from SRS and SHS groups (*n* = 3) and sequenced using Illumina HiSeq 2500 sequencer (Illumina Inc., USA). Briefly, total RNA samples were enriched by hybridization to bead-bound rRNA probes using Ribo Zero kit to obtain rRNA-depleted samples. This was followed by the construction of Illumina libraries using ScriptSeq v2 (Illumina Inc., USA) that applied unique barcode adapters. The libraries were assessed for their quality using Qubit dsDNA High Sensitivity DNA kit and Agilent 2100 Bioanalyzer (Agilent Technologies, A, USA; Agilent High Sensitivity DNA kit). This was followed with further enrichment and amplification of the libraries by qRT-PCR using KAPA Biosystems Library Quantification kit, and normalisation to 2nM. Equal volumes of individual libraries were pooled and run on a MiSeq using MiSeq Reagent kit v2 (Illumina) to validate the library clustering efficiency. The libraries were then re-pooled based on the MiSeq demultiplexing results and sequenced on a HiSeq 2500 sequencing platform (Illumina, San Diego, California, USA) and cBot with v3 flow cells and sequencing reagents. The library reads of greater than 30 to 35 million were generated for each individual library. The data were then processed using RTA and CASAVA thus providing four sets of compressed FASTQ files per library. All raw reads were pre-processed for quality assessment, adaptor removal, quality trimming and size selection using the FASTQC toolkit to generate quality plots for all read libraries. The RNA-Seq alignment and data analysis were all performed in-house using a high-performance computer; “Hydra”. The pipeline made use of Bash and Python scripting to accept RNA-Seq post-trimmed data as input, before ultimately producing output tables of differentially expressed transcripts. Paired-end (2X100bp) raw input data are initially aligned with Tophat to the sixth iteration of the *Rattus norvegicus* reference genome (Rn6) (Trapnell, Pachter & Salzberg, 2009). HTseq was used to generate read counts, using the ENSEMBL GRCh37 annotation for reference (Anders, Pyl & Huber, 2015). In the present study the pipeline used EdgeR statistical method from the R Bioconductor package to call differential gene expression (DGE) (Risso et al., 2014; Robinson, McCarthy & Smyth, 2010). This allowed us to predict low *p*-values (*p* < 0.05) and rank from highest to lowest fold changes (FC) which were utilised in downstream validation. Raw FASTQ files can be found at https://www.ebi.ac.uk/ena/data/view/PRJEB35016. A gene catalogues that described putative changes in gene expression as a consequence of being fed with HS diet in SHRs SFO, SON, PVN, NTS and RVLM were generated. All significantly expressed gene data then filtered to obtain genes expressed at a level of at least 20 read counts in one of the SHS versus SRS comparisons. This followed with sorting the genes according to FC from highest to lowest which later allowed us to select 10 genes (5 in each high and low FC) in each brain regions for subsequent validation with qRT-PCR. As well as validating the SHRs RNA-Seq data, the effect of the HS diet in WKY rats was also included.

### Quantitative reverse transcription polymerase chain reaction (qRT-PCR)

The cDNA synthesis was performed using QuantiTect Reverse Transcription kit (Qiagen) using total input RNA of 75ng for SFO, 200 ng for SON and PVN; 300 ng for NTS and 100 ng for RVLM, in accordance with manufacturer’s instructions. Primers for qRT-PCR were designed from NCBI official website (http://www.ncbi.nlm.nih.gov). All primers for target and endogenous control genes were obtained from Integrated DNA Technologies and the primer sequences are provided in Table S1. The qRT-PCR reactions were carried out in duplicate in 96-well plates and each PCR sample consisted of 6 µl 2X SYBR green master mix buffer (Roche), 0.024 µl of both forward and reverse primers 25nmole and 3.953 µl of RNase-free water. The reactions were performed using Applied Biosystems StepOne Plus Real-time PCR system and FCs were assessed by establishing delta-delta cycle threshold (C_T_) between *Rpl19*, a 60-s ribosomal protein L19 as a calibrator gene and target genes. The following temperature profile was used: two minutes at 95 °C for reverse transcription according to the manufacturer’s instruction, followed by 40 cycles at 95 °C for five seconds and 60 °C for 10 s. The average C_T_ values of target and calibrator genes obtained from qRT-PCR instrumentation were imported into a Microsoft Excel spreadsheet and the ΔΔC_T_ was calculated using (C)_TTarget_ - C_T__*Rpl*19_ as described by Livak & Schmittgen (2001).

**Figure 1:**
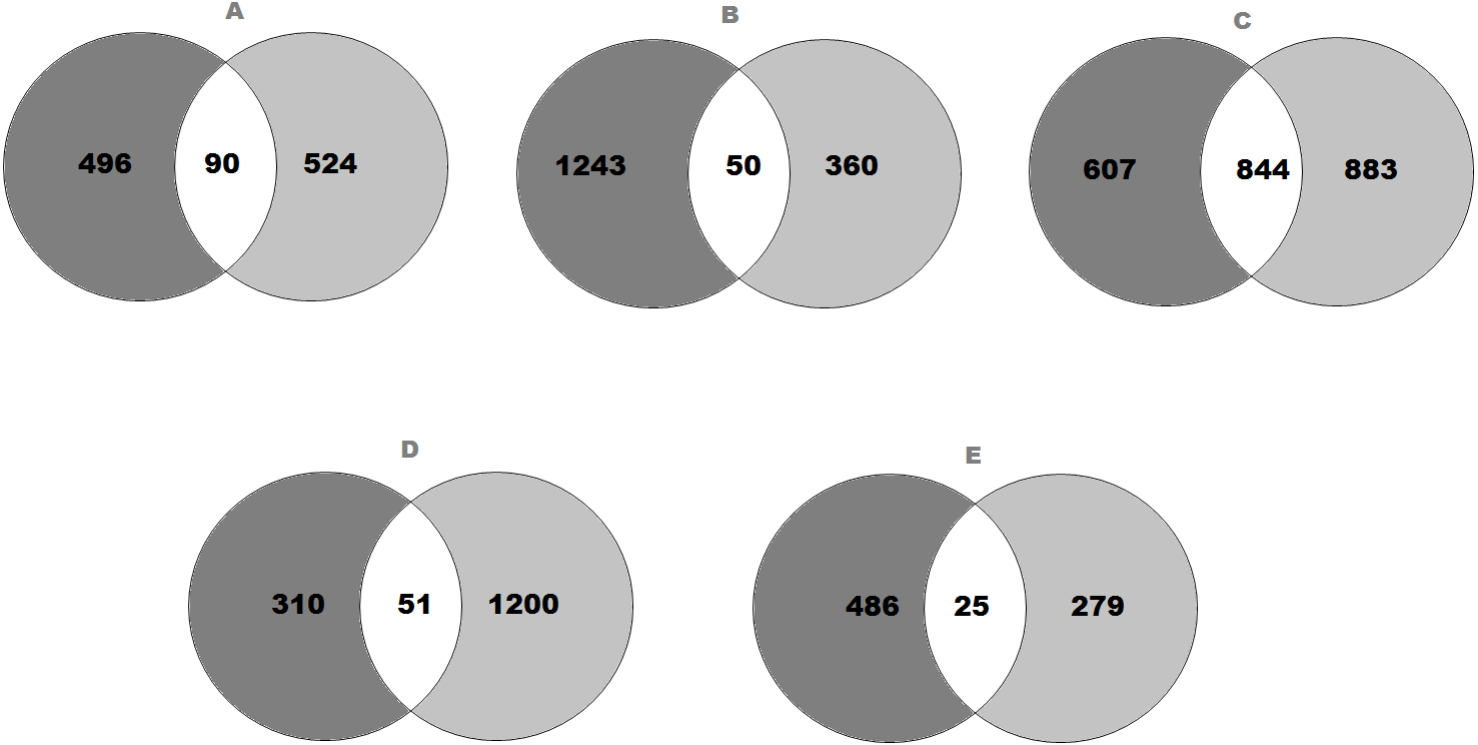
Venn diagram representing the number of genes that expressed significant changes in (A) SFO; (B) SON; (C) PVN; (D) NTS and (E) RVLM. The light grey area indicates (A) 586 genes in SFO; (B) 1,293 genes in SON; (C) 1,451 genes in PVN; (D) 361 genes in NTS and (E) 511 genes in RVLM of SHRs consuming high-salt (SHS) when compared with SHRs on regular-salt (SRS) diet; whilst the dark grey area represents (A) 614 genes in SFO; (B) 410 genes in SON; (C) 1,727 genes in PVN; (D) 1,251 genes in NTS and (E) 304 genes in RVLM of WKY rats on HS (WHS) when compared with WKY rats on RS (WRS) diet. Meanwhile, the (A) 90 genes in SFO; (B) 50 genes in SON; (C) 844 genes in PVN; (D) 51 genes in NTS and (E) 25 genes in RVLM are unique genes found in both strains of rats to intersect.

### Statistical analysis

Statistical analysis was performed using GraphPad Prism (GraphPad Software, La Jolla, CA, USA). All data are expressed as the mean ± standard error of means (SEM) of 6 rats. Comparisons between groups i.e., HS and RS of each individual strains were performed by independent unpaired Student’s *t*-test. The differences were considered statistically significant at *p* values <0.05.

## Results

### RNA-Seq analyses of SFO, SON, PVN, NTS and RVLM of WKY rats and SHRs fed with RS and HS diets

The transcriptomic data composed of gene catalogues with a wide variety of genes of diverse functions for all brain regions that compared the effect of high-salt (HS, 4% NaCl) diet with regular-salt (RS, 0.2% NaCl) diet in SHRs of SFO (Table S2), SON (Table S3), PVN (Table S4), NTS (Table S5) and RVLM (Table S6). All the significantly expressed genes of all brain regions are summarized in a Venn diagram (Figs. 1A to 1E). The Venn diagram explains the change in brain regions of SHRs fed with HS compared with SHRs fed with RS diet and similarly for WKY rats. In addition, the diagram also demonstrates the intersection between WKY rats and SHRs indicating the presence of genes in both strains of rats as consequence of HS diet intake.

In SFO, there were 586 genes found to be significantly changed (edgeR; *p* < 0.05) in SHRs fed with HS (4% NaCl) diet when compared with SHRs on RS (0.2% NaCl) diet. Meanwhile, there were 614 genes significantly changed in WKY rats receiving HS diet as compared to WKY rats on RS diet. As shown in Fig. 1A, 90 genes observed at the intersection, indicating expression changes of identical genes in SFO in SHRs (SHS compared to SRS) and WKY rats (WHS compared to WRS).

On the other hand, the expression levels of 1,293 genes were significantly (edgeR; *p* < 0.05) changed in SHRs fed with HS (4% NaCl) diet when compared with SHRs on RS (0.2% NaCl) diet. Meanwhile, 410 genes were found to be significantly changed in WKY rats receiving HS diet as compared to WKY rats on RS diet. As shown in Fig. 1B, 50 genes were observed in the intersection, indicating expression changes of identical genes in SFO in SHRs (SHS compared to SRS) and WKY rats (WHS compared to WRS).

Meanwhile, there were 1,451 genes showed significant changes (edgeR; *p* < 0.05) in their expression levels in SHRs fed with HS (4% NaCl) diet when compared with SHRs on RS (0.2% NaCl) diet in PVN. A comparison of WKY rats receiving HS diet with WKY rats fed with RS diet showed that the expression levels of 1,727 genes were significantly changed, as displayed in Fig. 1C. The same figure shows an intersection of 844 genes, indicating expression changes of identical genes in PVN in SHRs (SHS compared to SRS) and WKY rats (WHS compared to WRS).

In the NTS, 361 genes were found to be significantly changed (edgeR; *p* < 0.05) in SHRs fed with HS (4% NaCl) diet when compared with SHRs on RS (0.2% NaCl) diet. Meanwhile, 1,251 genes were significantly changed in WKY rats receiving HS diet as compared to WKY rats on RS diet. As shown in Fig. 1D, 51 genes observed at the intersection, indicating expression changes of identical genes in NTS in SHRs (SHS compared to SRS) and WKY rats (WHS compared to WRS).

Meanwhile, there were 511 genes showed significant changes (edgeR; *p* < 0.05) in their expression levels in SHRs fed with HS (4% NaCl) diet when compared with SHRs on RS (0.2% NaCl) diet in RVLM. A comparison of the WKY rats receiving HS diet with WKY rats fed with RS diet, showed that the expression levels of 304 genes were significantly changed, as displayed in Fig. 1E. The same figure shows an intersection of 25 genes, indicating expression changes of identical genes in RVLM in SHRs (SHS compared to SRS) and WKY rats (WHS compared to WRS).

### qRT-PCR validations of selected genes

The validation analyses for all target genes were compared relative to *Rpl19* as in all brain regions, *Rpl19* expression showed no significant differences in the expression when compared with that of another two well-known calibrator genes, *Gapdh* and *β-actin*, between the four experimental groups (WRS, WHS, SRS and SHS). For each tissue, based on the qRT-PCR analysis, validated genes were categorised as follows:

(A) Genes upregulated in both strains of rat.

(B) Genes downregulated in both strains of rat.

(C) Genes that showed significant upregulation only in SHRs.

(D) Genes that showed significant downregulation only in SHRs.

**Figure 2:**
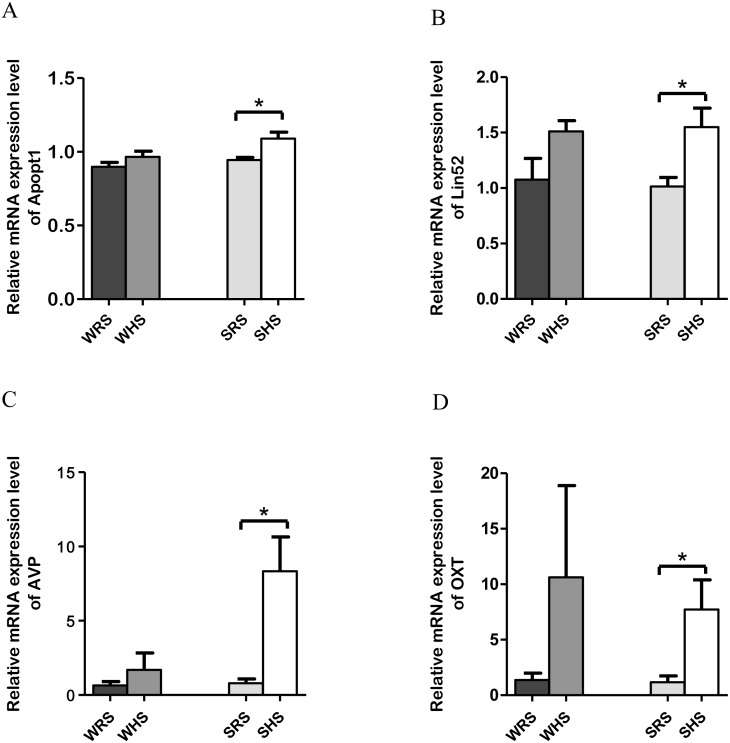
Relative mRNA expression levels of up-regulated genes i.e., *Apopt1*, *Lin52*, *AVP* and *OXT* only in SHRs of SFO. Data are presented as mean ± SEM; *n* = 6 rats. ^∗^*p* < 0.05 compared between SHS with SRS using Student’s *t*-test.

*SFO*: Among the 10 genes of the SFO chosen for validation (Table 1), 4 (*Apopt1*, *Lin52*, *AVP*, and *OXT*) were significantly upregulated only in SHRs when analyzed by qRT-PCR as evidenced in Figs. 2A to 2D.

**Table 1:** Summary of validation of putative SFO differentially expressed genes.

**Gene**	**Gene name**	**RNA-Seq analysis**				**qRT-PCR validation**
		**SHS. AvgCount**	**SRS. AvgCount**	**EdgeR.FC**	**EdgeR *P*-value**	**Category**
*Rmrp*	RNA component of mitochondrial processing endoribonuclease	13,845.20	5,339.07	2.58	2.02E–12	
*Crip1*	Cysteine-rich protein 1	226.25	149.41	2.53	6.15E–03	
*Hbb*	Homoglobin subunit β-1	5,450.44	2,198.55	2.47	2.90E–04	
*Lin52*	Protein Lin52	179.77	72.05	2.46	1.42E–03	C
*Apopt1*	Apoptogenic protein 1	258.84	105.15	2.45	1.32E–07	C
*AVP*	Vasopressin	127.89	12,256.18	0.01	1.20E–04	C
*Sim1*	Protein Sim 1	110.85	1,308.32	0.04	7.40E–04	
*Pdyn*	Proenkephalin-B	141.82	1,791.44	0.05	9.30E–04	
*OXT*	Oxytocin	219.56	3,452.25	0.06	1.00E–02	C
*Th*	Tyrosine 3-monooxygenase	57.55	504.04	0.07	2.39E–03	

**Notes.**

Category C: upregulated genes only in SHRs when analyzed with qRT-PCR.

Abbreviation Avg count average count of the genes’ number FC fold change *p*-value the sum of significance SHS SHRs fed with HS diet SRS SHRs fed with RS diet

**Figure 3:**
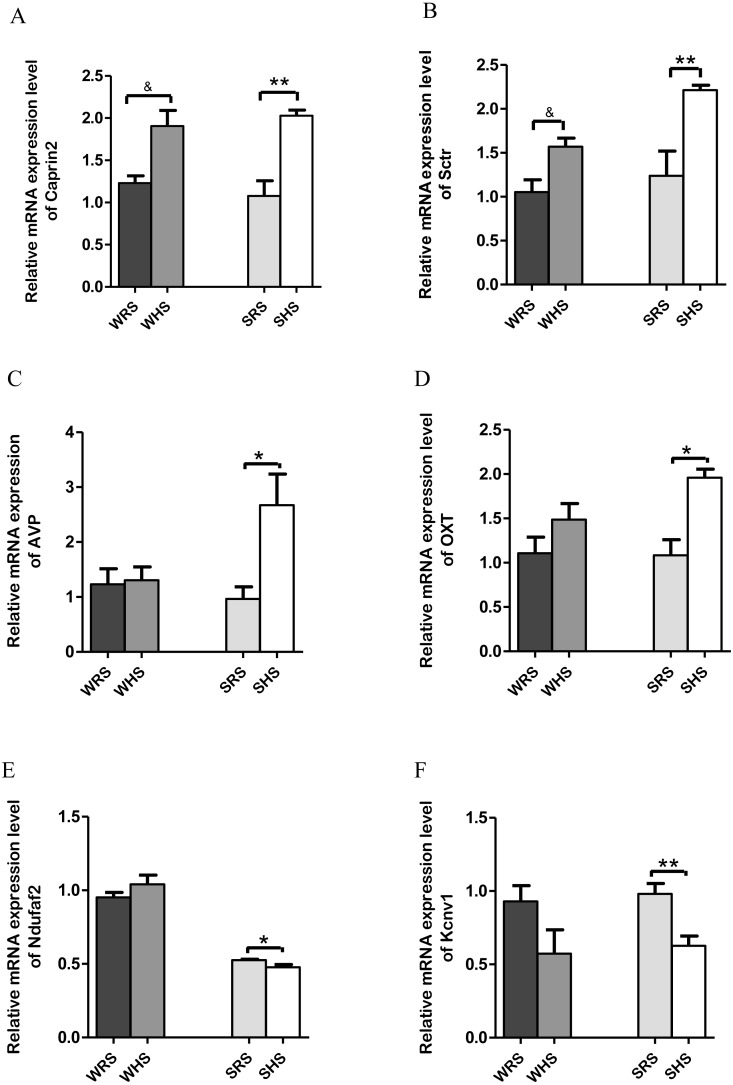
Relative mRNA expression levels of up-regulated genes i.e., (A) *Caprin2* and (B) *Sctr* in both SHRs and WKY rats; up-regulated genes i.e., (C) *AVP* and (D) *OXT* only in SHRs; and down-regulated genes i.e., (E) *Kcnv1* and (F) *Ndufaf2* only in SHRs. Data are presented as mean ± SEM; *n* = 6 rats. *p < 0.05 and **p < 0.01 compared between SHS with SRS and & *p* < 0.05 compared between WHS with WRS using Student’s *t*-test.

*SON*: Among the 10 genes of the SON chosen for validation (Table 2), 2 (*Caprin2* and *Sctr*) were significantly upregulated in both strains of rats (Figs. 3A and 3B); 2 (*AVP* and *OXT*) were significantly upregulated only in SHRs (Figs. 3C and 3D) and 2 (*Kcnv1* and *Ndufaf2*) were significantly downregulated only in SHRs (Figs. 3E and 3F) when analyzed by qRT-PCR.

**Figure 4:**
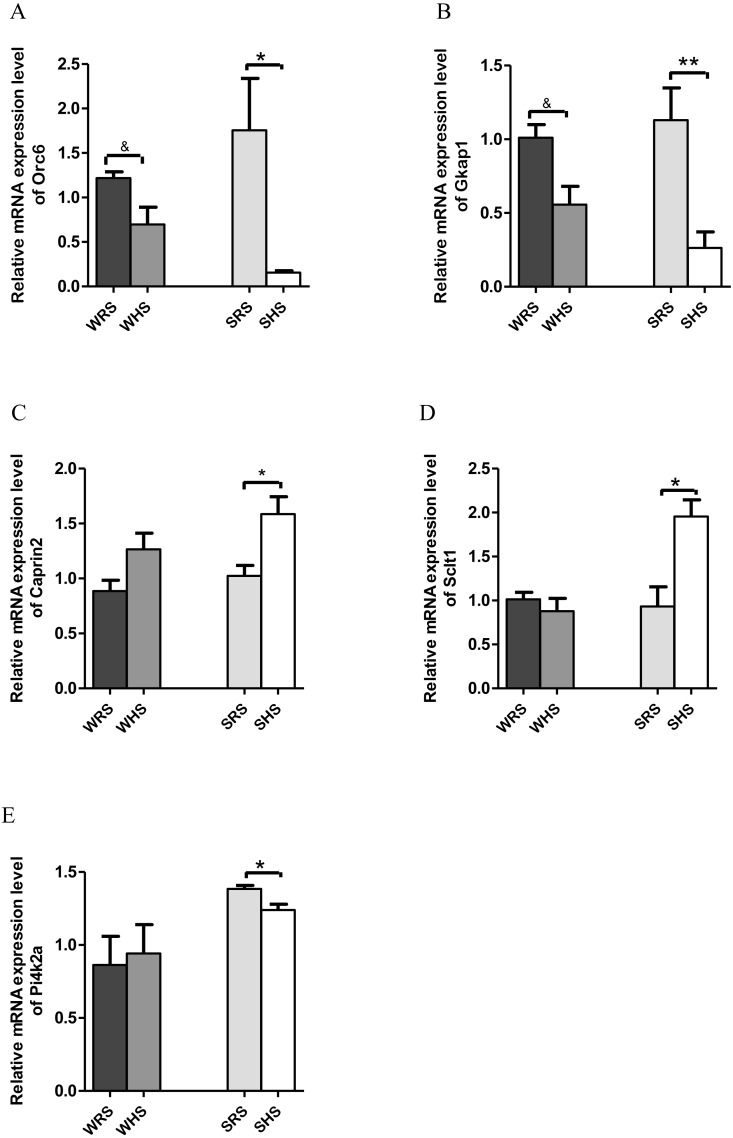
Relative mRNA expression levels of down-regulated genes i.e., (A) *Orc* and (B) *Gkap1* in both SHRs and WKY rats; up-regulated genes i.e., (C) *Caprin2* and (D) *Sclt* only in SHRs and down-regulated gene i.e., (E) *Pi4k2a* only in SHRs. Data are presented as mean ± SEM; *n* = 6 rats. ^∗^*p* < 0.05 and ^∗∗^*p* < 0.01 compared between SHS with SRS. *p < 0.05 and **p < 0.01 compared between SHS with SRS; and & *p* < 0.05 compared between WHS with WRS using Student’s *t*-test.

*PVN*. Among the 10 genes of the PVN chosen for validation (Table 3), 2 (*Orc6* and *Gkap*) were significantly downregulated in both strains of rats (Figs. 4A and 4B); 2 (*Caprin2* and *Sclt1*; Figs. 4C and 4D) were significantly upregulated only in SHRs and 1 (*Pi4k2a*) was significantly downregulated only in SHRs (Fig. 4E) when analyzed by qRT-PCR.

**Table 2:** Summary of validation of putative SON differentially expressed genes.

**Gene**	**Gene name**	**RNA-Seq analysis**				**qRT-PCR validation**
		**SHS. AvgCount**	**SRS. AvgCount**	**EdgeR.FC**	**EdgeR P-value**	**Category**
*OXT*	Oxytocin	35,909.30	62,162.60	1.73	1.13E–05	C
*Sctr*	Secretin receptor	159.86	137.71	1.58	4.24E–02	A
*Ndufaf2*	NADH: Ubiquinone oxidoreductase complex assembly factor 2	737.97	540.03	1.37	3.20E–04	D
*AVP*	Vasopressin	17,611.80	23,547.60	1.34	6.07E–03	C
*Caprin2*	Caprin family member 2	4,400.93	3,478.99	1.27	5.54E–03	A
*Kl*	Klotho	146.45	1,578.87	0.08	3.37E–04	
*Clic6*	Chloride intracellular channel 6	177.25	625.57	0.28	6.71E–03	
*Lrp1b*	LDL receptor related protein 1B	703.17	748.28	0.43	5.38E–01	
*Rgs7bp*	Regulator of G-protein signalling 7 binding protein	210.56	371.92	0.57	2.97E–03	
*Kcnv1*	Potassium-voltage gated channel modifier subfamily V member	94.46	138.54	0.68	1.77E–02	D

**Notes.**

Category A: upregulated genes in SHRs and WKY rats; Category C: upregulated genes only in SHRs and Category D: downregulated genes only in SHRs when analyzed with qRT-PCR.

Abbreviation Avg count average count of the genes’ number FC fold change *p*-value the sum of significance SHS SHRs fed with HS diet SRS SHRs fed with RS diet

**Figure 5:**
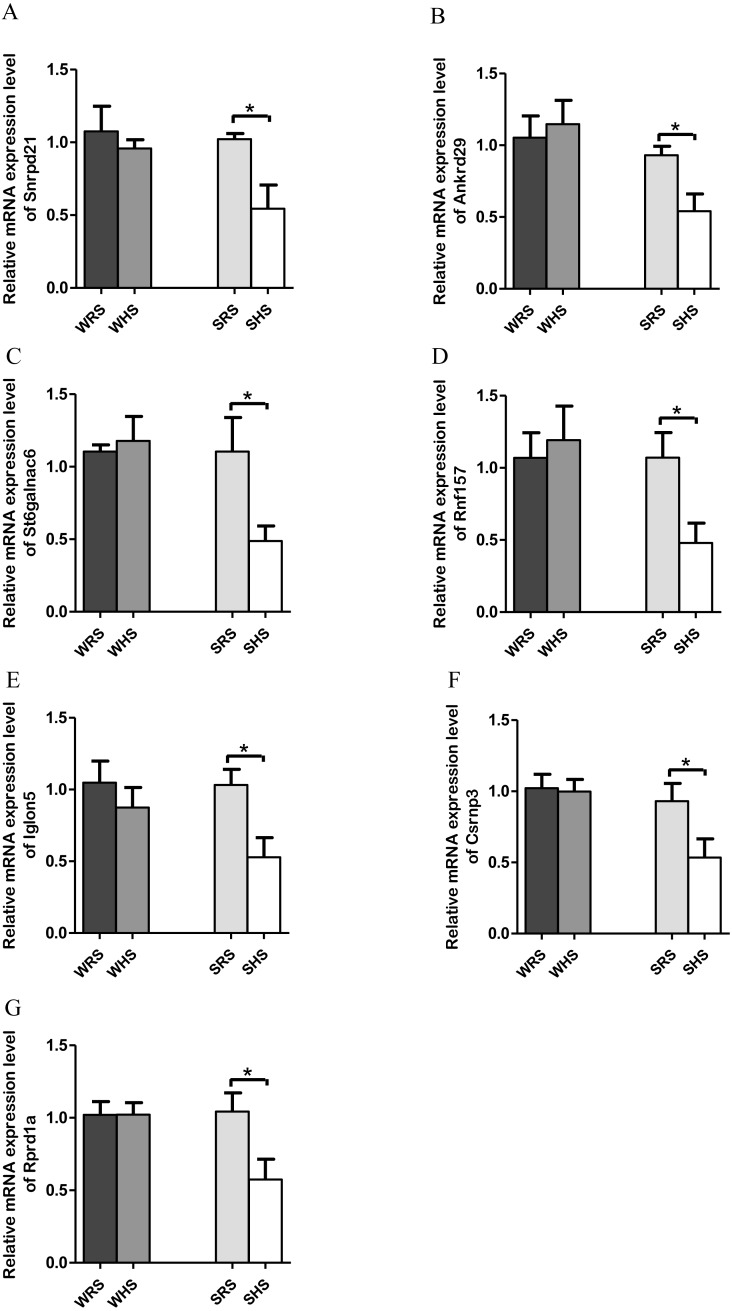
Relative mRNA expression levels of down-regulated genes i.e., (A) *Snrpd2l*, (B) *Ankrd*, (C) *St6galnac6*, (D) *Csrnp3*, (E) *Iglon5*, (F) *Rnf157*, and (G) *Rprd1a* only in SHRs. Data are presented as mean ± SEM; *n* = *D*6 rats. *p < 0.05 compared between SHS with SRS using Student’s *t* -test.

*NTS*. Among the 10 genes of the NTS chosen for validation (Table 4), 7 (*Rprd1a*, *Csrnp3*, *Snrpd2l*, *Iglon5*, *Rnf157*, *St6galnac6* and *Ankrd*) were significantly downregulated only in SHRs when analyzed by qRT-PCR as evidenced in Figs. 5A to 5G.

**Figure 6:**
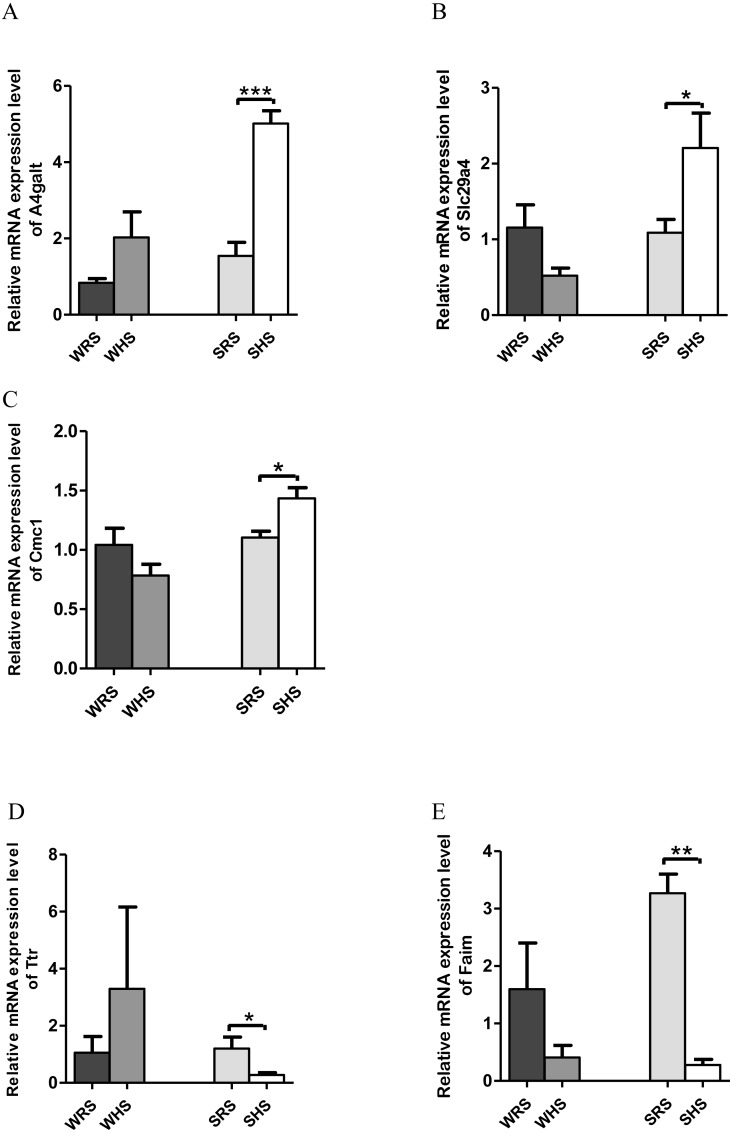
Relative mRNA expression levels of up-regulated genes i.e., (A) *A4galt*, (B) *Slc29a4* and (C) *Cmc1* only in SHRs; and down-regulated genes i.e., (D) *Ttr* and (E) *Faim* only in SHRs. Data are presented as mean ± SEM; *n* = 6 rats. *p < 0.05, **p < 0.01 and ***p < 0.001 compared between SHS with SRS using Student’s *t*-test.

*RVLM*: Among the 10 genes of the RVLM chosen for validation (Table 5), 3 (*A4galt*, *Slc29a4* and *Cmc1*) were significantly upregulated only in SHRs (Figs. 6A–6C) whilst 2 (*Ttr* and *Faim*) were significantly downregulated only in SHRs (Figs. 6D to 6E) when analyzed by qRT-PCR.

## Discussion

### General consideration

In the present study, RNA-Seq was used to document changes in gene expression profile in SFO, SON, PVN, the hypothalamic forebrain region for maintaining homeostasis; and NTS and RVLM brainstem CV control centres in SHRs following the consumption of an HS diet. The SHR is a well-documented animal experimental model in functional genetic and physiological studies as it has been attributed to the similarity of its pathophysiology with essential HPN in human (Doggrell & Brown, 1998; Korner, 2010; Leong, Ng & Jaarin, 2015). Several expert panels have reported that SHRs are an excellent model of experimental HPN that could serve as a counterpart for clinical essential HPN as well as model for complications of HPN (Badyal, Lata & Dadhich, 2003). Moreover, it has been proposed that the pathogenesis of HPN in the SHR is heterogeneous with the underpinning of CNS, neurohumoral, renal, and cellular abnormalities (DePasquale et al., 1992; Fortepiani et al., 2003; Sarikonda et al., 2009). Our previous findings showed significant augmentation in mean arterial pressure (MAP) of SHRs fed with HS diet when compared with SHRs on a regular-salt diet. However, there was no significant change in the MAP of WKY rats as a consequence of the consumption of HS-diet. Thus, further confirming that SHRs developed a salt-sensitive component to the established HPN and are a model for studying the central mechanisms of salt-sensitive HPN.

### RNA-Seq analysis and qRT-PCR validation

Using the SHR model, catalogues of genes that are differentially expressed in SFO, SON, PVN, NTS and RVLM as a consequence of consuming the HS diet were generated using RNA-Seq analysis which was then validated with qRT-PCR. We also asked if the HS differentially expressed genes validated in the SHRs were also altered in expression by HS in normotensive WKY rats’ SFO, SON, PVN, NTS and RVLM. The comparisons were made between HS and RS diets in both the rat strains that revealed four categories of genes: genes up-regulated (A) or down-regulated (B), in both SHRs and WKY rats; genes up-regulated only in SHRs (C); genes down-regulated only in SHRs (D). Here, one might assume that it is the latter genes (categories C and D) that might have importance in the elevated BP seen in this strain as a consequence of HS. However, it may well be that genes commonly regulated in both strains have different effects due to WKY rats- or SHRs-specific interactions dependent upon genetic background. We also concede that we have revealed a relatively very small number of HS-responsive genes and many more remain to be mined from our RNAseq datasets.

#### Subfornical organ (SFO)

The SFO is known to be an important CV control region regulating fluid homeostasis by initiating volumetrically controlled thirst and drinking responses (Denton et al., 1999; Freece et al., 2005; Smith, Beninger & Ferguson, 1995; Stachenfeld, 2008). The osmotic information through osmoreceptors from OVLT is transmitted neurally to the hypothalamus and ultimately results in thirst sensation, drinking behaviour and release of AVP; hence, retain water in the body. Thus, the up-regulation of *AVP* in the present study (Fig. 2C) is in accordance to the claim that the rat’s SFO to have high-affinity binding sites for AVP as well as its degradation product of AVP (Anthes et al., 1997; Jurzak, Fahrenholz & Gerstberger, 1993). Furthermore, the mammalian SFO has been reported to contain vasopressinergic fibre endings from magnocellular or parvocellular portions, and the presence of *AVP* mRNA in SFO implies that AVP is formed and released there (Anthes et al., 1997). On the other hand, *OXT* was shown to be expressed in a similar pattern as *AVP*. Both AVP and OXT have been reported to be secreted simultaneously in response to hyperosmolality and hypovolemia (Haanwinckel et al., 1995; Ventura et al., 2002), and they have synergistic effect on the inner medullary collecting ducts (Ventura et al., 2002). The oxytocinergic fibres have also been reported to be found in SFO suggesting that OXT may affect salt and water intake in rats (Hosono et al., 1999). As such, the up-regulation of *OXT* level in SFO of SHRs in the present study is defensible.

**Table 3:** Summary of validation of putative PVN differentially expressed genes.

**Gene**	**Gene name**	**RNA-Seq analysis**				**qRT-PCR validation**
		**SHS. AvgCount**	**SRS. AvgCount**	**EdgeR.FC**	**EdgeR *P*-value**	**Category**
*Pi4k2a*	Phosphatidylinositol 4-kinase Type 2 alpha	232.15	108.97	2.13	4.27E–06	D
*Prrc2a*	Proline rich coiled-coil 2A	960.03	509.31	1.89	1.38E–06	
*AVP*	Vasopressin	9,331.38	5,415.72	1.73	8.45E–09	
*Caprin2*	Caprin family member 2	2,337.86	1,551.92	1.51	4.57E–04	C
*OXT*	Oxytocin	37,526.70	28,121.00	1.23	1.86E–03	
*Sclt1*	Sodium Channel and Clathrin Linker 1	1,663.35	1,707.38	0.97	8.46E–01	C
*Gkap1*	G Kinase Anchoring Protein 1	1,417.12	2,419.13	0.59	2.80E–05	B
*Orc6*	Origin Recognition Complex Subunit 6	97.98	173.50	0.57	3.29E–02	B
*Isy1*	SY1 Splicing Factor Homolog	498.23	915.38	0.55	1.67E–05	
*Nsmce1*	NSE1 Homolog, SMC5-SMC6 Complex Component	125.59	235.92	0.54	2.77E–03	

**Notes.**

Category B: downregulated genes in SHRs and WKY rats; Category C: upregulated genes only in SHRs and Category D: downregulated genes only in SHRs when analyzed with qRT-PCR.

Abbreviation Avg count average count of the genes’ number FC fold change *p*-value the sum of significance SHS SHRs fed with HS diet SRS SHRs fed with RS diet

**Table 4:** Summary of validation of putative NTS differentially expressed genes.

**Gene**	**Gene name**	**RNA-Seq analysis**				**qRT-PCR validation**
		**SHS. AvgCount**	**SRS. AvgCount**	**EdgeR.FC**	**EdgeR P-value**	**Category**
*Nkain4*	Sodium/Potassium Transporting ATPase Interacting 4	165.64	110.68	1.50	1.38E–02	
*St6galnac6*	ST6 N-Acetylgalactosaminide Alpha-2,6-sialyltransferase 6	638.48	436.19	1.47	2.83E–03	D
*Ankrd29*	Ankyrin Repeat Domain 29	161.05	142.31	1.50	4.96E–02	D
*Rnf157*	Ring Finger Protein 157	912.69	673.41	1.36	3.67E–02	D
*Rprd1a*	Regulation of Nuclear Pre-mRNA Domain Containing 1A	695.73	899.43	0.77	3.70E–02	D
*Csrnp3*	Cysteine and Serine Rich Nuclear Protein 3	980.12	1,302.83	0.75	2.56E–02	D
*Pdyn*	Prodynorphin	488.66	753.66	0.65	5.09E–03	
*Snrpd2l*	Small Nuclear Ribonucleoprotein D2-like	199.17	377.78	0.53	5.68E–03	D
*Romo1*	Reactive Oxygen Species Modulator 1	159.40	119.00	3.06	2.43E–06	
*Iglon5*	IgLON Family Member 4	128.26	155.44	0.51	4.71E–03	D

**Notes.**

Category D: downregulated genes only in SHRs when analyzed with qRT-PCR.

Abbreviation Avg count average count of the genes’ number FC fold change *p*-value the sum of significance SHS SHRs fed with HS diet SRS SHRs fed with RS diet

In addition, both RNA-Seq and qRT-PCR data also revealed significant up-regulation of *Lin52* and *Apopt1* (Table 1 and Figs. 2A and 2B) in SHRs being fed with HS diet. The *Lin52* (protein Lin52) which was significantly up-regulated in SHS has been associated with clinical CV events among African Americans from atherosclerosis risk communities’ study. A genome-wide analysis of meta-analysis study by Shendre et al. (2017) showed the presence of *Lin52* gene in African-American of CV events such as stroke, atherosclerosis and myocardial infarction incidents. However, the study did not further evaluate the functional role of this gene with the occurring of these diseases and suggested further studies. Hence, our finding may propose that HS intake could be the initial triggering factor leading to CV diseases. However, its certainty needs further exploration. Meanwhile, the *Apopt1*, an apoptogenic protein 1, has been shown to be localised within mitochondria and related with mitochondrial disorders characterised by cyclooxygenase (COX) deficiency (Melchionda et al., 2014). The COX deficiency is one of the most common biochemical abnormalities found in mitochondrial disorders with half of all cases remain genetically undefined. Moreover, a connection between apoptosis and increased reactive oxygen species (ROS) production has been suggested to play an important role in the pathophysiology of mitochondrial diseases associated with COX deficiency (Di Giovanni et al., 2001; Kadenbach et al., 2004; Melchionda et al., 2014). Therefore, the expression of *Apopt1* in SFO needs further work to elucidate its functional significance in salt-induced HPN.

#### Supraoptic nucleus (SON)

The SON is known as a specialised nucleus in the hypothalamus that contains magnocellular neurons that secrete only AVP and OXT (Armstrong, 1995). A large amount of AVP and OXT are released into bloodstream to play a critical role in the regulation of salt and water balance as well as acting as vasoconstrictor, antidiuretic, CV regulator, lactation and affiliative behaviour when the normal physiological osmolality and volume are challanged (Hatton, 1990; Hatton, 1997; Jansen, Van der Zee & Gerkema, 2007; Mlynarik et al., 2007; Young, 1999). Thus, the high rates of neuropeptide synthesis, transport, and release in AVP and OXT have made SON as an important experimental model for the study peptidergic neuronal cell biology (Burbach et al., 2001; Hatton, 1990). The present RNA-Seq and qRT-PCR analyses of SON showed up-regulation of *AVP* and *OXT* (Table 2 and Figs. 3C and 3D) genes in SHRs as a result of consuming HS. These findings are in accordance with many other previous studies that have shown large increases in expression of the principal neuropeptide genes, *AVP* and *OXT* in magnocellular neurons (MCNs) in SON during salt-loading (Burbach et al., 2001; Greenwood et al., 2015b; Johnson et al., 2015c). It has been claimed that chronic salt loading produces large increase in volumes of MCNs which are recognized to be due to global increase in transcription and protein synthesis in the MCNs under hyperosmotic stimulations (Johnson et al., 2015c; Miyata, Nakashima & Kiyohara, 1994; Zhang et al., 2001). In addition, the increased *AVP* level in SHRs is in accordance with the hypothesis that SHRs have high AVP release i.e., high transcription of *AVP* as increasing levels of mineralocorticoid receptor and mineralocorticoids have been regarded to be involved in central regulation of BP, and they are known to drive the release of AVP to maintain hypertension (Carmichael & Wainford, 2015; Pietranera et al., 2012). Hence, it is not a surprise to observe a drastic increase of *AVP* in SHRs fed HS but there remains a need to elucidate the mechanism of how AVP related to salt-sensitive HPN. The increased expression of *AVP* is associated with an increased expression of *Caprin2*. The *Caprin2* is a gene that encodes RNA binding protein that plays a role in central osmotic defense receptor (Konopacka et al., 2015; Loh et al., 2017). It was found to directly bind to mRNA that encodes *AVP* and responsible to increase the length of poly-A tail (structures added to the end of all newly-made mRNAs as more *AVP* mRNAs molecules needed to produce during dehydration) (Konopacka et al., 2015). The team also concluded that *Caprin2* plays a critical role in mediating brain responses to osmotic stress as it is expressed in MCNs in SON and PVN, and that *Caprin2* expression in these neurones increases during osmotic stress. The study further demonstrated that *Caprin2* knockdown in SON and PVN disrupts physiological osmoregulatory mechanisms. Therefore, the up-regulation of *Caprin2* in the present study (Fig. 3A) further strengthen the findings of studies by Konopacka et al. (2015). On the other hand, the OXT which is known to be co-secreted with AVP in response to osmotic and blood volume changes also been reported to induce a decrease in MAP and total peripheral resistance (Dutil et al., 2001; Petersson et al., 1996). This further suggest that OXT may play an important role in the control of body fluids homeostasis, causing natriuresis and vasodilatation, and reducing cardiac contractility and heart rate. Thus, the upregulation of *OXT* in SHRs fed with HS (Fig. 3D) suggesting that *OXT* along with *AVP* is transcribed more than normal levels induced by HS diet in SHRs in order to maintain BP.

**Table 5:** Summary of validation of putative RVLM differentially expressed genes.

**Gene**	**Gene name**	**RNA-Seq analysis**				**qRT-PCR validation**
		**SHS. AvgCount**	**SRS. AvgCount**	**EdgeR.FC**	**EdgeR P-value**	**Category**
*A4galt*	Alpha 1,4-galactosyltransferase	150.17	122.08	2.32	2.62E–03	C
*Pnmt*	Phenylethanolamine-N-methyltransferase	159.04	71.17	2.27	2.51E–03	
*Snrpd2l*	Small Nuclear Ribonucleoprotein D2-like	426.26	214.49	1.99	4.60E–02	
*Slc29a4*	Solute Carrier Family 29 Member 4	425.29	242.05	1.77	2.75E–03	C
*Gmfb*	Glia Maturation Factor, Beta	1,271.81	735.76	1.75	7.62E–03	
*Ttr*	Transthyretin	237.20	1,103.01	0.22	1.35E–02	D
*Gmfg*	Glia Maturation Factor, gamma	125.93	178.24	0.34	1.68E–03	
*Cmc1*	C-x(9)-C Motif containing 1	82.97	206.42	0.41	1.65E–07	C
*S100a6*	S100 Calcium Binding Protein A6	130.77	262.70	0.50	3.29E–03	
*Faim*	Fas Apoptotic Inhibitory Molecule	278.34	386.77	0.73	3.80E–02	D

**Notes.**

Category C: upregulated genes only in SHRs and Category D: downregulated genes only in SHRs when analyzed with qRT-PCR.

Abbreviation Avg count average count of the genes’ number FC fold change *p*-value the sum of significance SHS SHRs fed with HS diet SRS SHRs fed with RS diet

In addition, the *Sctr*, a secretin receptor is known as a potent regulator of pancreatic bicarbonate, electrolyte and volume secretions (Baiocchi et al., 1999; Chow et al., 2004; Chu et al., 2007) was found to up-regulated in both SHRs and WKY rats fed with HS diet (Fig. 3Aii). The secretin though originally isolated from upper intestinal mucosal extract, has been associated with translocation of water channels such as translocation of aquaporin 2 triggered by AVP and OXT to and from plasma membrane in renal tubules, a critical role for renal water absorption (Chu et al., 2007; Jeon et al., 2003; Noda & Sasaki, 2005). Since AVP has been proved in regulating renal water reabsorption and some of these mechanisms have been associated with cyclic-AMP-protein kinase A-mediated phosphorylation of water channels, it has been hypothesized that secretin could modulate renal water permeability by inducing AVP-independent translocation of functional aquaporin 2 (Chu et al., 2007; Jeon et al., 2003; Li et al., 2006; Lorenz et al., 2003; Wilke et al., 2005) which the water homeostasis is very much related to salt-sensitive HPN.

On the other hand, *Ndufaf2* and *Kcnv1* (Figs. 3E and 3F) were found to be down-regulated in SHRs fed with HS diet. Mutation of *Ndufaf2* has been reported in mitochondrial related diseases in humans (Anderson et al., 2013; Chinnery & Hudson, 2013; Koene et al., 2012); however, its exact role pertaining to HPN has not been documented. Meanwhile, the *Kcnv1*, voltage-gated channels have been reported as one of the gene expressed in preeclampsia, a hypertensive condition in pregnant women (Chang et al., 2011). To the best of our knowledge, the information of this gene associating with SSH is still lacking and not well documented in animal models.

#### Paraventricular nucleus (PVN)

The PVN is an important central sympathoexcitatory region which may become more active in hypertensive conditions (Allen, 2002; Geraldes et al., 2014a). It has been also referred to as a command nucleus providing feedforward excitatory synaptic drives to coordinate lower brainstem CV and respiratory motor activity (Dampney et al., 2005). The activation of PVN promotes an increase in sympathetic output and a pressor effect mediated via direct and indirect projections via RVLM to the spinal cord thereby accessing sympathetic neurons to modulate BP (Badoer, 2001; Coote, 2005; Hardy, 2001; Motawei et al., 1999). Extensive studies have been conducted even at the molecular level which all indicated PVN as an important hypothalamus region in BP modulation. The present study is also to serve added knowledge to the existing findings on PVN.

Similar, to SON, the PVN also accounts for greater secretion of AVP with clear cardinal physiological function i.e., to control renal excretion of water, to regulate hemodynamic parameters dependent of effective blood volume (vasopressor activity) and to regulate secretion of ACTH (from PVN’s subdivision of parvocellular neurons) (Sherman et al., 1986). It is also well documented that the hypothalamic levels of *AVP* mRNA increase following chronic salt-loading and/or dehydration (Greenwood et al., 2015a; Hayashi et al., 2006; Holbein, Bardgett & Toney, 2014; Holbein & Toney, 2015), and this is clearly indicated in the present study which the mRNA expression level of *AVP* in PVN was increased in the expression insignificantly in SHRs fed with HS diet (Table 3). The SHRs is known for overactivated sympathetic activity even before hypertension development (Simms et al., 2009); thus, several increased in FC in *AVP* mRNA expression in chronic exposure to salt diet is considered as an estimated outcome. On the other hand, the increase in the expression of *OXT* in SHRs (Table 3) as a consequence of HS diet further strengthen the importance of OXT in salt appetite. In addition, it is known that OXT is released along with AVP upon hypertonic stimulation. Meanwhile, the *Caprin2* (Fig. 4C) was also found to express in a similar manner as SON for the reason explained earlier. In addition to *AVP* and *OXT*, the PVN also expressed other genes with multiple functions. The *Sclt1* (sodium channel and clathrin linker 1) has been found to be associated with nephronophthisis, the most frequent genetic cause of chronic renal failure in children (Failler et al., 2014). This gene was found to be up-regulated in SHRs fed with HS diet; however, the relative contribution of this gene to salt-sensitive HPN is not able to explain at this moment.

On the other hand, *Pi4k2a* (Fig. 4E), *Gkap1* and *Orc6* (Figs. 4A and 4B) were found to be down-regulated in SHRs fed with HS diet. The *Pi4k2a*, mRNA encoding phosphatidylinositol 4-kinase type 2 isoform A is found in synaptic vesicles and involved in exocytosis which is important in the release of neurotransmitter from synaptosomes (Solich et al., 2015). Pharmacological inhibition of *Pi4k2a* causes significant decrease in norepinephrine release but does not affect the release of GABA or glutamate, suggesting an association of *Pi4k2a* with norepinephrine neurons (Khvotchev & Sudhof, 1998; Solich et al., 2015). A study conducted by Solich and team (2016) showed that *Pi4k2a* was down-regulated in the frontal cortex of norepinephrine transporter (NET) knock-out mice. The NET has been claimed to serve as the main target of antidepressant drugs and many diseases, and HPN has been linked with NET dysfunction (Bonisch & Bruss, 2006). Furthermore, the activity of NET is dependent on the concentration of sodium/chloride ions (Shafqat et al., 1993) and it present has been detected in brain regions rich with norepinephrine terminals i.e. thalamus, hypothalamus and amygdala (Bonisch & Bruss, 2006; Lorang, Amara & Simerly, 1994; Schroeter et al., 2000). Hence, the downregulation of this gene in SHRs being fed with HS diet seem to corelate with the development salt-sensitive HPN. Furthermore, this gene was also found in kidney transcriptome as well as haemoglobin where both studies were related to HPN (Marques et al., 2017; Zhang et al., 2014), and it has also been reported that *Pi4k2a* act via WNT-signaling pathway (Kuzmanov et al., 2016).

#### Nucleus tractus solitarii (NTS)

The NTS plays a pivotal role in the regulation of both the set-point of BP (Waki et al., 2010; Waki, Takagishi & Gouraud, 2014) and the gain of baroreflex for homeostatic control of BP (Waki et al., 2010). It is the principal site of termination of baroreceptor afferent fibres and as such mediates inhibitory action of baroreceptor on sympathetic discharge (Frigero, Bonagamba & Machado, 2000; Machado, 2001; Talman, Perrone & Reis, 1981; Waki, Takagishi & Gouraud, 2014). This area contains many neurotransmitters or neuromodulators that are important in CV control, and the intermediate portion of NTS is richly innervated by fibres arising from different brain nuclei that are also known to have an important role in CV control (Colombari et al., 2001; Duale et al., 2007; Kasparov & Teschemacher, 2008; Zoccal et al., 2014). The NTS neuron sends excitatory amino acid projections to the CVLM which in turn, inhibits RVLM neuron via GABAergic inhibitory pathway (Guyenet, 2006b; Sapru, 1996; Yao et al., 2008).

The present qRT-PCR data revealed that HS diet altered the expression level of genes that are functionally associated with transporters, transmembrane, signalling pathways, transcription factors and binding protein in both NTS. Genes such as *St6galnac6*, *Akrd29*, *Rnf157*, *Rprd1a*, *Csrnp3*, *Snrpd1a,* and *Iglon5* were found significantly down-regulated only in SHRs fed with HS diet (Figs. 5A and 5G). The downregulation of *Snrpd2l*, small nuclear ribonucleoprotein D2-like suggests that aspects of RNA processing are changed in NTS due to HS diet. Meanwhile, the *Csrnp3* has been reported as one of the targeted genes in causing HPN (Miao et al., 2017; Yamamoto et al., 2013); however, the functional role of it was not discussed. The *Rnf157*, a ring finger protein 157, has been described to predominantly be expressed in brain and is implicated in the regulation of neuronal survival (Matz et al., 2015). Thus, its downregulation in the present study remains to be elucidated. Similarly, to *St6galnac6*, ST6 N-acetylgalactosaminide alpha-2,6-sialyltransferase 6 which was identified as an enzyme in the synthesis of disialyl monosialyl Lewis (Tsuchida et al., 2003). Meanwhile, the *Ankrd2a*, ankyrin repeat domain 2a has been identified to belongs to the family of stress-inducible protein that expressed in striated muscle and its mutation has been recognised in cardiomyopathy patients (Bang et al., 2014). Finding by Jasnic-Savovic et al. (2015) has suggested *Ankrd2a* silencing to alter hypertrophic and dilated cardiomyopathy pathways and demonstrated the localisation of this protein in the intercalated disk of human cardiomyocytes. As there were not many reports on the presence of this gene in animal models, it hints for detail exploration in them. Meanwhile, *Rprd1a* has been associated with Wnt/β-catenin signalling and considered as an inhibitor for cell proliferation (Liu et al., 2015; Zhang et al., 2012). In addition, a study by Arvind et al. (2015) has reported up-regulation of *Rprd1a* in coronary artery disease in human. However, the correlation of this gene with SSH is yet to be documented and our study may be considered to be the first to report of its existence induced by HS diet. Similarly, the *Iglon5* which its functional role remains to be elucidated.

#### Rostral ventrolateral medulla (RVLM)

The RVLM is the region where the sympathetic pre-motor neurons controlling vasomotor sympathetic nerve activity are located. Thus, it is important in determining the basal sympathetic tone and receives inputs from various autonomic areas within the central nervous system (Kumagai et al., 2012; Thomas et al., 2013). The RVLM neurons project to the sympathetic preganglionic neurons in the IML cell column of the spinal cord and receive a direct glutamatergic projection from NTS (Card et al., 2006; Dampney, 1994; Geraldes et al., 2014b; Leman, Viltart & Sequeira, 2000). These neurons also receive excitatory and inhibitory inputs from other brain areas, such as the hypothalamus and other part of the medulla oblongata (Koga et al., 2008; Sved, Ito & Yajima, 2002). The RVLM has been found to have enhanced angiotensin-II-dependent superoxide accumulation under the influence of dietary salt (Braga, 2010).

In the present qRT-PCR analysis, *A4galt*, *Slc29a4*, and *Cmc1* were found to be up-regulated in SHRs being fed with HS diet. The *Slc29a4*, a nucleosides transporter, which is known for its presence in renal epithelia (Duan & Wang, 2013) with minimal functional property has been reported to be catalyse the reuptake of monoamines into presynaptic neurons, thus determining the intensity and duration of monoamine neural signalling. In addition, it has also been shown to transport several compounds, including serotonin, dopamine and neurotoxin 1-methyl-4 phenylpyridinium. If this is the case, the upregulation of this gene in SHRs (Fig. 6B) may have been partially resolved; however, further studies are still require to ascertain the functional properties pertaining to HS diet intake. Meanwhile, *A4galt* has been reported to be up-regulated in clinical trials of primary aldosteronism (Chenlong et al., 2017), the most common form of endocrine hypertension which has been characterized by excessive and autonomous aldosterone secretion causing increased sodium retention, potassium excretion, hypervolemia, suppressed renin activity and HPN (Rossi, 2011). Therefore, the upregulation of *A4galt* in the present study (Fig. 6A) may suggest a direct or indirect involvement in increasing MAP in SHRs being fed with the HS diet.

Meanwhile, *Ttr* and *Faim* were found to be significantly down-regulated in SHRs (Figs. 6D and 6E), where *Ttr* mutation was strongly associated in amyloidosis, a genetic disorder characterized in many forms such as amyloid cardiomyopathy amyloid, familial amyloid polyneuropathy, leptomeningeal amyloidosis (Faria et al., 2015; Gertz et al., 2015; Gonzalez-Lopez et al., 2015; Lobato et al., 2003; Palaninathan, 2012) and senile amyloidosis (typical pulmonary lesions) (Kruczak et al., 2013). The amyloidosis has been linked with the occurrence of HPN as reported in case of studies by Eder et al. (2007), Culafic et al. (2007) and Cirulis et al. (2016); however, all these reports failed to mention on the mechanism of *Ttr* in regulating HPN.

## Conclusions

In summary, high dietary sodium intake is thought to be one of the most prevalent risk factors for hypertension in modern societies, and there is a need to better understand the mechanisms involved. We found that chronic ingestion of a HS diet altered gene expression profiles at the level of SFO, SON, PVN, NTS and RVLM in both SHRs and WKY rats. These findings are suggestive of changes in hypothalamic forebrain and brainstem signalling systems that might participate in SSH. We suggest that, irrespective of the primary cause of hypertension, the genes involved in generating and regulating CV autonomic outflow from the hypothalamic forebrain and brainstem are potential targets for effective new therapies for SSH.

##  Supplemental Information

10.7717/peerj.8528/supp-1 Table S1 Dataset

10.7717/peerj.8528/supp-2 Table S2 RNA-Seq datasets for SFO

10.7717/peerj.8528/supp-3 Table S3 RNA-Seq datasets for SON

10.7717/peerj.8528/supp-4 Table S4 RNA-Seq datasets for PVN

10.7717/peerj.8528/supp-5 Table S5 RNA-Seq datasets for NTS

10.7717/peerj.8528/supp-6 Table S6 RNA-Seq datasets for RVLM

10.7717/peerj.8528/supp-7 Supplemental Information 1 qPCR Raw data: SFO

10.7717/peerj.8528/supp-8 Supplemental Information 2 qPCR Raw Data: SON

10.7717/peerj.8528/supp-9 Supplemental Information 3 qPCR Raw Data: PVN

10.7717/peerj.8528/supp-10 Supplemental Information 4 qPCR Raw Data: NTS

10.7717/peerj.8528/supp-11 Supplemental Information 5 qPCR Raw Data: RVLM

## Additional Information and Declarations

### Data Availability

The following information was supplied regarding data availability: The raw data is available at EBI: PRJEB35016.
